# Heterostructured mixed metal oxide electrocatalyst for the hydrogen evolution reaction

**DOI:** 10.3389/fchem.2023.1141361

**Published:** 2023-03-14

**Authors:** Dwi Sakti Aldianto Pratama, Andi Haryanto, Chan Woo Lee

**Affiliations:** Department of Chemistry, Kookmin University, Seoul, Republic of Korea

**Keywords:** hydrogen evolution reaction, electrocatalyst, mixed metal oxide, reaction mechanism, modulation strategy

## Abstract

The hydrogen evolution reaction (HER) has attracted considerable attention lately because of the high energy density and environmental friendliness of hydrogen energy. However, lack of efficient electrocatalysts and high price hinder its wide application. Compared to a single-phase metal oxide catalyst, mixed metal oxide (MMO) electrocatalysts emerge as a potential HER catalyst, especially providing heterostructured interfaces that can efficiently overcome the activation barrier for the hydrogen evolution reaction. In this mini-review, several design strategies for the synergistic effect of the MMO catalyst on the HER are summarized. In particular, metal oxide/metal oxide and metal/metal oxide interfaces are explained with fundamental mechanistic insights. Finally, existing challenges and future perspectives for the HER are discussed.

## 1 Introduction

Net-zero emissions have been extensively discussed in the context of combating climate change (Dresp et al., 2019; Na et al., 2019; Khan et al., 2021). However, more than 80% of our energy demand is met by fossil fuels such as oil, coal, and gas (Ritchie et al., 2020). The development of green and clean energy sources, such as solar and wind energy, has been hindered because of their intermittent weather-dependent nature (Song et al., 2020). Hydrogen is as an alternative green energy source with a high energy density of 120 MJ kg^-1^, which exceeds that of its predecessor fuels such as oil, coal, and gas, and only H_2_O is produced as the combustion by-product (Hughes et al., 2021). However, hydrogen only accounts for 2.5% of global energy consumption, with 94 million tons (Mt) in demand in 2021. Only 1 Mt hydrogen is produced from low-carbon emission sources. In the future, 100 Mt of low-emission hydrogen is required to be produced annually to meet net-zero emissions by 2050 (IEA, 2022).

Sustainable hydrogen production *via* water electrolysis can be easily achieved by placing two electrodes in an aqueous electrolyte (Ifkovits et al., 2021; Raza et al., 2022). A sufficient voltage difference will cause water to split into its elemental components, with hydrogen (H_2_) being formed at the cathode and oxygen (O_2_) being formed at the anode. Depending on the electrolyte conditions, H_2_ can be formed through the reaction 2H^+^_(aq)_ + 2e^−^ → H_2(g)_ in acidic conditions or 2H_2_O+ 2e^−^ → H_2_ + 2OH^−^ in alkaline conditions. One factor that limits hydrogen production from water splitting is the sluggish kinetics and high thermodynamic energy barrier in the form of overpotential energy (Choi et al., 2021; Wang et al., 2021). Platinum is the best catalyst for the hydrogen evolution reaction (HER). However, its scarcity and high price limit its wide application. Hence, there is a need for developing electrocatalysts with materials that are more abundant and cheaper for the HER.

Metal oxides have been receiving significant attention from the research community owing to their compositional and structural diversity, flexible tunability, low cost, abundance, and environmental friendliness for use as electrocatalysts (Song et al., 2018; Zhu Y. et al., 2020). Despite its unique properties, pristine metal oxides remained unsatisfactory for practical application, especially in the HER, due to the unfavorable intermediate binding strength and stability issues (Cho et al., 2018; Adamson et al., 2020). Mixed metal oxides (MMOs) emerge as a new strategy to fully utilize metal oxides for HER electrocatalysts because they still inherit their unique pristine properties. Optimized adsorption energies can be obtained, and the intrinsic catalytic activity limit can be surpassed in heterostructured MMOs, resulting in a higher HER efficiency. (Lv et al., 2019; Suryanto et al., 2019). Also, the electrocatalysts’ stability in an aqueous environment and alkaline electrolyte was improved significantly (Bates et al., 2015; Cho et al., 2018). Compared to other mixed metal-based ceramic materials, heterostructured MMO is also one of the potential approaches as an affordable electrocatalyst for industrial applications due to its facile synthesis method and lower noble metal loading amount (Seuferling et al., 2021; Nie et al., 2022; Pagliaro et al., 2022). However, there is still no clear guideline for use of MMOs as HER electrocatalysts.

In this mini-review, recent advancements in heterogeneous heterostructured MMOs for HER electrocatalysts, characterized by their overpotential and Tafel slope values, are summarized. We start by elaborating the mechanistic understanding of the HER mechanism and then, summarize the achievement of heterogeneous heterostructured MMO electrocatalysts in the HER. Design strategies for heterostructured MMO electrocatalysts, such as metal oxide/metal oxide and metal/metal oxide heterojunctions, are also explained fundamentally. The synergistic effects of each heterostructured MMO’s composition, morphology, and structure with high HER performance are also fundamentally explained. Finally, we highlight the challenges faced in catalyst development and provide future directions for catalyst development.

## 2 Reaction mechanisms

**FIGURE 1:**
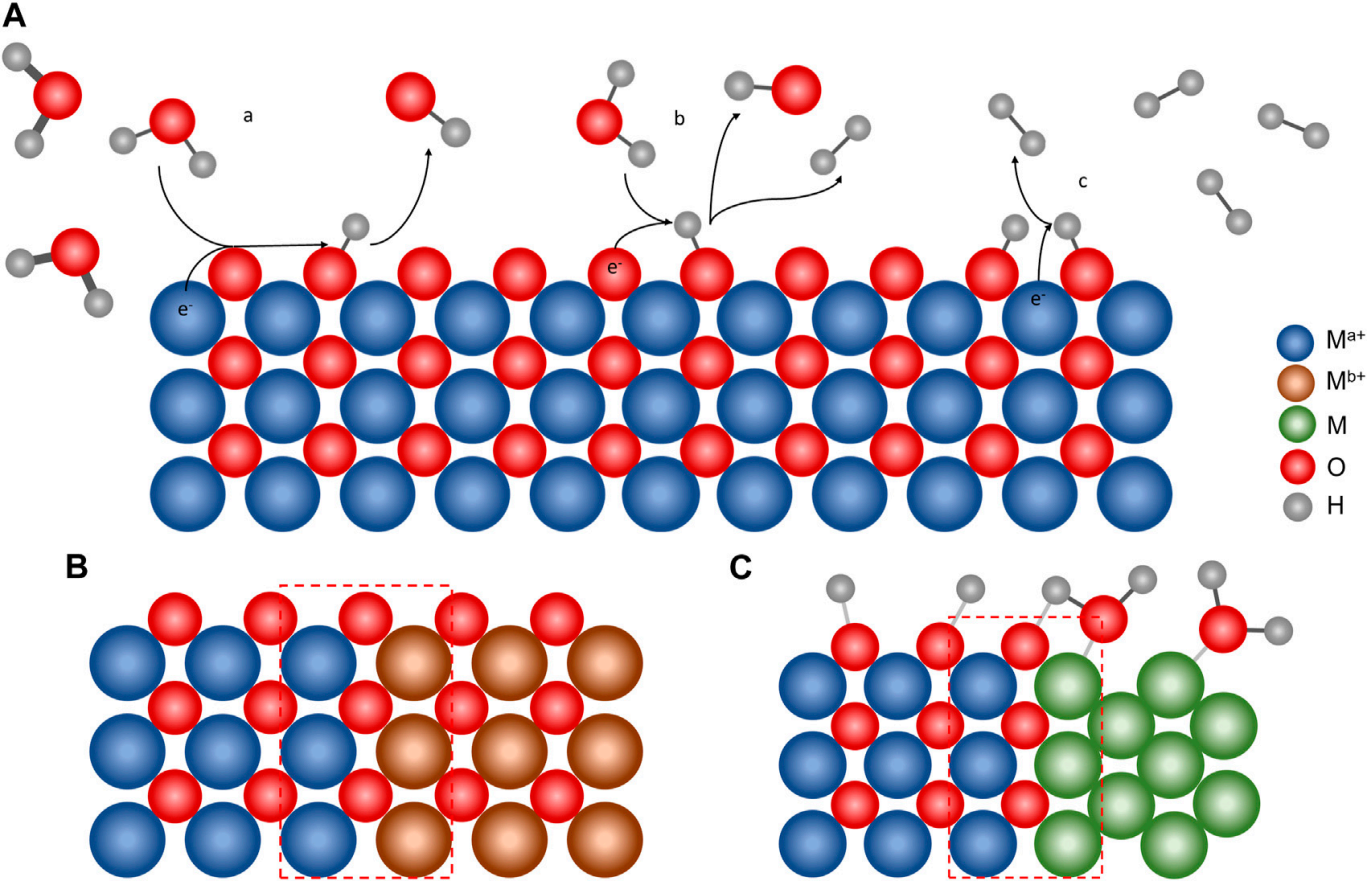
**(A)** Mechanism of the HER on a metal oxide surface, with (a) Volmer, (b) Heyrovsky, and (c) Tafel steps. Material strategies to increase the HER performance by heterostructured MMOs: **(B)** metal oxide/metal oxide and **(C)** metal/metal oxide interfaces. The heterojunction shown in red dashed lines plays an important role in enhancing the HER activity of the heterostructured MMO.

The HER occurs *via* a multistep electrochemical process (i.e., Volmer, Heyrovsky, and Tafel steps) that involves a two-electron transfer and takes place on the catalyst surface, as shown in Figure 1A. In an acidic medium, the hydronium ion (H_3_O^+^) acts as a proton source, and the HER proceeds as follows:

$${H_{3}O}^{+}+e^{-}+M\to M-H+H_{2}O\;\left(Volmer\right),$$ (1)

$$M-H+{H_{3}O}^{+}+e^{-}\to H_{2}+H_{2}O+M\;\left(Heyrovsky\right),$$ (2)

$$2\;M-H\to 2\;M+H_{2}\;\left(Tafel\right).$$ (3)

A proton is adsorbed on the catalyst’s vacant site, which is represented by M, and an M–H bond is formed (Eq. 1). Then, hydrogen formation can occur in two ways: the dissociation of the M–H bond and H^+^ transfer from another H_3_O^+^ in the bulk solution (Eq. 2) or the dissociation of two M–H bonds (Eq. 3). The proton can originate from H_2_O in an alkaline or neutral medium, and in this case, the Volmer and Heyrovsky steps changed as follows:

$$H_{2}O+e^{-}+M\;\to \;M-H+{OH}^{-}\;\left(Volmer\right),$$ (5)

$$M-H+H_{2}O+e^{-}\to \;H_{2}+{OH}^{-}+M\;\left(Heyrovsky\right).$$ (6)

Dissociation of H_2_O is critical for M–H bond formation in an alkaline or neutral medium. Breaking the H–O–H bond to provide a proton requires higher energy than breaking the H_3_O^+^ ion, which readily gives a proton in an acidic medium. Therefore, the HER rate in an alkaline or neutral medium is lower than that in an acidic medium (Hao et al., 2018; Nie et al., 2022; Zhang et al., 2023).

It is well-known that the Tafel slope is closely related to the HER mechanism. Suppose the Tafel slope is about 120 mV dec^−1^, the Volmer step is the rate-determining step (RDS), while the values of around 40 and 30 mV dec^−1^ correspond to Heyrovsky and Tafel steps, respectively. If the Volmer step is the RDS, hydrogen coverage on the electrode surface should be 0 because almost all of the adsorbed hydrogen will be consumed rapidly in the Heyrovsky and Tafel steps. By contrast, the Heyrovsky step-limiting reaction has nearly constant full hydrogen coverage on the electrode surface (Bao et al., 2021).

## 3 Current progress on mixed metal oxide electrocatalysts for the HER

Heterostructured MMOs have been used as electrocatalysts in the HER. The performance of heterostructured MMOs, such as the overpotential and Tafel slope values, is listed in Table 1. Previous studies have shown that MMOs have high HER activity, close to the Pt/C catalyst, known as the HER catalyst benchmark (Cho et al., 2018; Wang et al., 2022). This section discusses several strategies for developing heterogeneous MMO electrocatalysts categorized by their metal oxide/metal oxide interface and metal/metal oxide interface (Figures 1B, C).

**TABLE 1:** Overview of MMO electrocatalyst materials for the HER under acidic, alkaline, and neutral environment.

Catalyst	Preparation method	Electrolyte	Overpotential at 10 mA cm^-2^ (mV)	Tafel slope (mV dec^−1^)	References
RuO_2_/NiO/NF	Hydrothermal method followed by chemical etching	1 M KOH	22	32	Liu et al. (2018)
Ni–Fe nanoparticle	Metal-micelle formation followed by thermal reduction	1 M KOH	100	58	Suryanto et al. (2019)
B-TiZr-2.5	Sol–gel method followed by Mg–H_2_ reduction method	0.5 M H_2_SO_4_	160	87	Singh et al. (2020)
Ni/CeO_2_-CNT	Solvothermal method followed by thermal reduction	1 M KOH	91	N/A	Weng et al. (2015)
Co_3_O_4_/CeO_2_ heterostructure	Solvothermal method followed by annealing	1 M KOH	88	48	Zhang et al. (2021)
NiO/CeO_2_ heterostructure	Solvothermal method followed by annealing	1 M KOH	99	60	
NiFe LDH	Hydrothermal synthesis	1 M PBS	87	48.4	Yuan et al. (2019)
Er_2_Si_2_O_7_:IrO_2_ (cube)	Sol–gel method	0.5 M H_2_SO_4_	130	49	Karfa et al. (2018)
		2 M PBS	190	67	
		1 M KOH	170	59	
NiO/Co_3_O_4_	Precipitation followed by calcination	1 M KOH	170	119	Wei et al. (2019)
Ni_3_(VO_4_)_2_@NiCo_2_O_4_/NF	Two-step hydrothermal method	1 M KOH	113	101	Shang et al. (2017)
RuO_2_/Co_3_O_4_	Sol–gel method	1 M KOH	89	91	Liu et al. (2017)
Ir_0.8_Ru_0.2_O_y_	Electrospinning	1 M NaOH	30	32	Cho et al. (2018)
CoO/Fe_3_O_4_	Annealing (thermal reduction in Ar gas flow)	1 M KOH	220	73	Adamson et al. (2020)
Ru–CeO_2_/C	Wet chemical reduction	0.1 M KOH	100	68	Pagliaro et al. (2022)
NiCu MMO	Wet chemical reduction	1 M KOH	200	120	Faid et al. (2021)
NiCu 3D network	3D printing followed by thermal oxidation	1 M KOH	70	67	Zhou et al. (2021)
MoPt/Ti_0.9_Ir_0.1_O–C	Hydrothermal synthesis followed by chemical reduction	0.5 M H_2_SO_4_	21	24	Pham et al. (2023)
NiTe_x_/MoO_y_/NiMoO_4_	Solvothermal	1 M KOH	59	33	Xu et al. (2023)
PdZn/TiO_2-x_ nanosheets	Hydrothermal synthesis followed by the impregnation process	1 M KOH	64	68	Naik et al. (2023)
NiWO_4_@NiCoO_x_S_y_/NCF	Three-step hydrothermal process	1 M KOH	85	76	Yang et al. (2023c)
Ru/Cu–Cu_2_O@C	Metal–organic framework followed by chemical impregnation	1 M KOH	26	29	Liu et al. (2023b)
Co_3_(PO_4_)_2_-MoO_3–*x*_/CoMoO_4_/NF	Hydrothermal synthesis followed by phosphatization	1 M KOH	24	24	Yang et al. (2023a)
Mo–NiO/Ni nanopores	Dealloying process	1 M KOH	34	49	Pang et al. (2023a)
ZnCo_2_O_4_@CoMoO_4_	Two-step hydrothermal process	1 M KOH	114	114	Yang et al. (2023b)
Ni_4_Mo/MoO_2_/C	Plasma-enhanced chemical vapor deposition	1 M KOH	77	100	Wei et al. (2023)
NiCo_2_O_4_@C12NF	Atomic layer deposition followed by the hydrothermal process	1 M KOH	96	51	Pang et al. (2023b)
Ru@V–RuO_2_/C	Thermal annealing followed by low-temperature oxidation	0.5 M H_2_SO_4_	46	55	Li et al. (2023)
		1 M KOH	6	45	
PtNb–Nb_2_O_5_	Microwave-assisted synthesis	0.5 M H_2_SO_4_	21	22	Nie et al. (2022)
		Neutral seawater	225	56	
		1 M KOH	28	26	
PtNi@CeO_2_	Solvothermal	1 M KOH	59	56	Liu et al. (2023a)

### 3.1 Metal oxide/metal oxide interface

**FIGURE 2:**
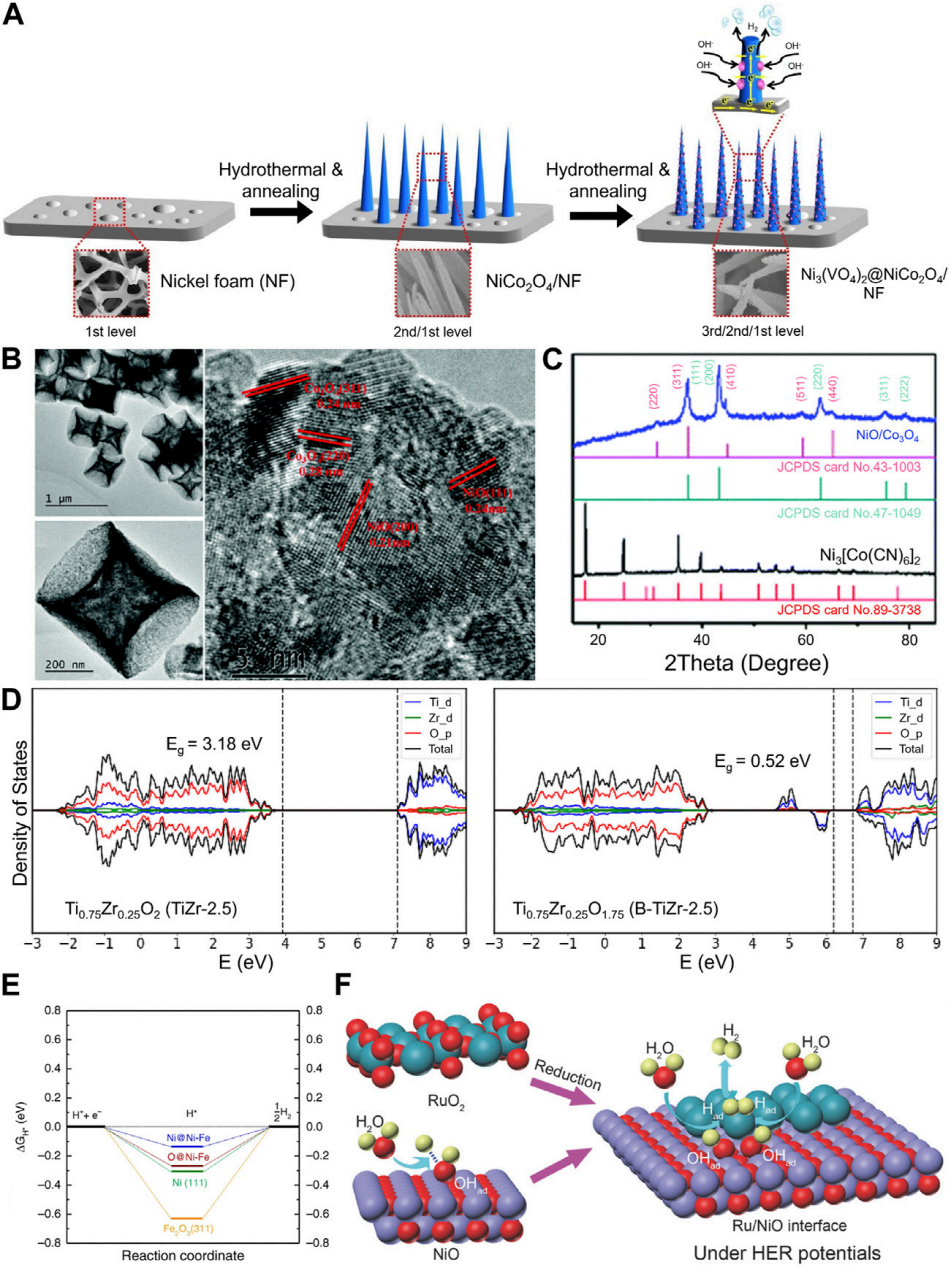
**(A)** Schematic diagram of the steps involved in the fabrication of a three-dimensional hierarchical structure of Ni_3_(VO_4_)_2_@NiCo_2_O_4_/NF. In the fabrication process, NiCo_2_O_4_ nanowires were grown on nickel foam by a hydrothermal process, and Ni_3_(VO_4_)_2_ was then grown on NiCo_2_O_4_ nanowires by a hydrothermal process reproduced with permission from Shang et al. (2017). **(B)** HRTEM image of the heterostructured NiO/Co_3_O_4_ concave microcuboid which shows individual lattices of Co_3_O_4_ and NiO. **(C)** Individual lattice was also supported by XRD measurement which shows NiO and Co_3_O_4_ peaks coexisted in the sample [reproduced with permission from Wei et al. (2019)]. **(D)** Density of states of Ti_0.75_Zr_0.25_O_2_ and partially reduced Ti_0.75_Zr_0.25_O_1.75_. Introducing oxygen vacancies can generate new electronic states, thereby lowering the bandgap [reproduced with permission from Singh et al. (2020)]. **(E)** Gibbs free energy diagram of the hydrogen adsorption and desorption on the Fe_2_O_3_(311) and Ni(111) surface and the Ni–Fe interface [reproduced with permission form Suryanto et al. (2019)]. **(F)** MMO-derived Ru–NiO interface showing the synergistic effect induced by the interface of RuO_2_-derived Ru and NiO interface under HER potentials [reproduced with permission from Liu et al. (2018)].

Transition metal oxides (TMOs) have been used as the HER electrocatalyst, yet single-TMO catalysts have been limited by their intrinsic activity. Electrocatalytic activity kinetics is closely related to reactant availability at the catalyst surface (Nguyen et al., 2020). Heterostructured MMOs utilized highly engineered morphology by overgrowing certain compounds on top of each other. One example is by growing Ni_3_(VO_4_)_2_@NiCo_2_O_4_ on nickel foam (NF) by using a two-step hydrothermal process. First, NF was used as a foundation to provide the macropores necessary for electrolyte penetration (micropore size: 300 µm). Then, one-dimensional structured NiCo_2_O_4_ was grown on the top of the NF. Last, Ni(VO_4_)_2_ was grown on the top of NiCo_2_O_4_ nanowires, as shown in Figure 2A. The catalyst showed high HER performance at the heterojunction between Ni_3_(VO_4_)_2_ and NiCo_2_O_4_ to obtain an overpotential of 113 mV at 10 mA cm^-2^ with a Tafel slope value of 101 mV dec^−1^ (Shang et al., 2017).

Later, it was found that the heterojunction on the metal oxide/metal oxide interface plays an important role in enhancing catalytic activity; the heterojunction in the heterostructured MMO has shown better HER activity than alloys or oxide composites. The heterojunction allowed MMOs to have a more exposed active site than alloys or oxide composites (Faid et al., 2021). To increase the number of heterojunctions to an atomic level, Wei and coworkers prepared NiO/Co_3_O_4_ concave surface microcubes from a metal–organic framework (MOF) precursor (Ni_3_[Co(CN)_6_]_2_) (Wei et al., 2019). The MOF precursor can ensure homogeneous distribution of NiO/Co_3_O_4_ on the cubes. High-resolution transmission electron microscopy (HRTEM) analysis shows a lattice of NiO and Co_3_O_4_ on the cube surface. It was also supported by X-ray diffraction (XRD) analysis, which shows that NiO and Co_3_O_4_ coexisted in the sample (Figures 2B, C). Furthermore, the concave surface obtained from step-by-step annealing allows for electrolyte penetration and increased surface area. The obtained catalyst allowed electrolyte penetration, which favored HER kinetics, and it showed an overpotential of 169.5 mV to achieve a 10 mA cm^-2^ current density and a Tafel slope of 119 mV dec^−1^.

Seuferling et al. (2021) showed that a wide compositional range of heterostructure metal oxide catalysts could be deposited easily on an electrode substrate by using a microwave-assisted method involving metal carbonate precursors. Microwave assistance provides sufficient heating for decomposing metal carbonates and facilitates a homogeneous distribution of amorphous oxides on the substrate. Among various amorphous metal oxides synthesized, Co_0.8_Ni_0.2_ deposited on NF showed the highest performance, with an overpotential of 350 mV being needed to achieve a current density of 100 mA cm^-2^ (Seuferling et al., 2021).

The electrocatalytic activity of a particular reaction is dependent on the binding intermediates, which determine the reaction kinetics and final product (Hao et al., 2018; Lee et al., 2018). The electronic structure of the catalyst surface governs the binding affinity for the energy of the reaction intermediates. Furthermore, electronic structure engineering modulates electron transport across the catalyst (Chandrasekaran et al., 2020). Hence, an appropriate design of the electronic structure can enhance electronic conductivity and increase charge transfer between the catalyst and electrolyte.

Similar to single-metal oxide electrocatalysts, oxygen vacancy (V_O_) engineering can be used to tailor the electronic structure of MMO electrocatalysts (Zhang et al., 2018; Hona et al., 2020, 2021). Singh et al. (2020) successfully improved the HER activity of a TiO_2_/ZrO_2_ composite by introducing oxygen vacancies into the catalyst to obtain an overpotential value of 160 mV at 10 mA cm^-2^ and a Tafel slope value of 87 mV dec^−1^. The grain boundary defects resulting from the introduced ZrO_2_ could create a charge imbalance on the heterostructure. As a result, an extra proton binds onto the oxygen atom associated with the charge imbalance, creating an excess of -OH groups on the surface, increasing surface acidity. Moreover, the density of state calculation showed that introducing V_O_ to the obtained composite heterostructure generates new electronic states, and the bandgap was reduced from 3.18 to 0.52 eV (Figure 2D), thus increasing the electronic conductivity of the catalyst.

Cerium oxide (CeO_2_) has shown potential electrocatalytic activity due to the flexible transition valence state between Ce^4+^ and Ce^3+^ (Weng et al., 2015; Xu et al., 2022). The idea to generate a TMO/CeO_2_ heterostructure rich in V_O_ has been employed by Zhang et al. (2021). The interaction between CeO_2_ and TMOs, such as Co_3_O_4_, can tune the electronic structure and reduce HBE. Furthermore, the coexistence of both Ce^3+^ and Ce^4+^, and also Co^2+^ and Co^4+^, could generate an abundance of V_O_ on the surface. Consequently, the Co_3_O_4_/CeO_2_ catalyst showed a high HER performance of 88 mV overpotential to achieve a 10 mA cm^-2^ current density and a Tafel slope of 48 mV dec^−1^.

Further optimization in the electronic structures can be achieved by creating multiple vacancies from a combination of the V_O_-modulated surface with metal vacancies. For example, Yuan et al. (2019) fabricated the NiFe layered double hydroxide with multiple vacancies. The formation of multiple vacancy defects, including oxygen and metal vacancies, leads to an increase in the electrochemical surface area (ECSA), smaller charge resistance, lower bandgap, and increased reaction kinetics. As a result, incredible HER performance was observed in a neutral solution of 87 mV to obtain a 10 mA/cm^−2^ with 46.3 mV dec^−1^ of the Tafel slope value (Yuan et al., 2019).

### 3.2 Metal/metal oxide interface

Yan and coworkers introduced an amorphous heterostructure catalyst for the HER and developed a three-dimensional core/shell nanosheet by chemically reducing Co_3_O_4_ nanosheets in a hydrogen atmosphere (Yan et al., 2015). The reduction in the hydrogen atmosphere results in the formation of a Co(100) core and a thin layer of amorphous cobalt oxide, which can be observed by HRTEM analysis. This unique structure can provide high electrical conductivity in the core, which acts as an electron reservoir, and high HER activity on the surface resulting from defects such as dangling bonds and oxygen vacancies on the surface. The structure showed an overpotential of 129 mV to drive a 20 mA cm^-2^ current density and a Tafel slope of 44 mV dec^−1^. Yet, prolonged HER activity performance will only result in a decrease of cobalt oxide due to electrochemical reduction.

Optimizing the H intermediate adsorption energy is the key to enhancing the HER activity. It can be achieved by delicately designing the catalyst interface (Kim et al., 2020). According to the Sabatier principle, the interaction between the catalyst and intermediate reaction should be just right, neither too strong, where the intermediates fail to desorb and block the active sites, nor too weak, in which the intermediates fail to bind to the catalyst (Zhu J. et al., 2020). Moreover, heterostructuring could promote single-metal oxide catalysts and reduce the noble metal loading amount (Kim et al., 2022). Therefore, introducing two components with different binding strengths on the catalyst interface would modify the binding strength, which would imitate the ideal catalyst.

Due to its nature, metal oxides often undergo electrochemical reduction during the HER (Adamson et al., 2020). Heterostructuring the Ni/NiO_x_ composite with TMOs such as Cr_2_O_3_ stabilizes Ni/NiO_x_ (Bates et al., 2015). Suryanto et al. (2019) also showed that Fe^3+^ in the Fe_2_O_3_ half-cell potential was altered due to the heterostructure. The Janus Ni–Fe_2_O_3_ nanoparticles were formed through ion-oleate metal surfactant complex (micelles) formation that facilitates adjacent iron oxides and metallic Ni. The heterogeneous interface of Ni(111) and Fe_2_O_3_(311) surfaces was connected *via* bridge O atoms. Density-functional theory (DFT) calculations showed that O atoms of Fe_2_O_3_ and Ni could act as H atom adsorption sites. Interestingly, bridge O atoms and Ni atoms at the interface had more optimal ΔG_H*_ values of −0.27 and −0.14 eV compared to −0.62 and −0.31 eV of Ni(111) and O atoms in the Fe_2_O_3_(311) site, respectively (Figure 2E). Consequently, the Janus Ni–Fe_2_O_3_ nanoparticles had a high HER activity with a low overpotential value of 46 mV at 10 mA cm^-2^ and a small Tafel slope of 58 mV dec^−1^ (Suryanto et al., 2019).

The formation of a heterostructure interface could help promote water dissociation in the water-splitting reaction. Two different compounds adjacent to each other, which have different binding strengths, could modulate water dissociation completely. Using this idea, Qiao’s group used NiO as a bifunctional promotor for RuO_2_ in the water-splitting reaction because of the strong M–OH bond affinity of NiO, which could promote HO–H cleavage (Figure 2F). The reduction of RuO_2_ species into metallic Ru under HER potential could facilitate hydrogen adsorption and recombination. Thus, the synergistic effect of Ru metal and NiO resulted in high HER performance with an overpotential of 22 mV at a current density of 10 mA cm^-2^ and a Tafel slope of 31.7 mV dec^−1^ (Liu et al., 2018).

Heterostructuring can generate a new way to break the intrinsic catalytic activity. Easier water dissociation is promoted by synergistic effects at the interfaces. The charge transfer induced from the interfaces enables weakened reactant adsorption, hence preventing surface poisoning from the bonded reactants (Janani et al., 2022; Surendran et al., 2022), resulting in easier hydrogen adsorption and desorption.

The morphological shape is an important factor to be considered for enhancing HER activity (Karfa et al., 2018). The edge and corner sites have a higher electric field distribution, which is conducive to easier charge transfer (Alfath and Lee, 2020). One-dimensional structured catalysts have caught the attention of the research community lately (Kuang et al., 2016; Liu et al., 2017). The strong electric field at the tip of the 1D structured catalyst allows higher charge transfer. Furthermore, the structure is believed to enhance bubble removal during the HER. Cho et al. (2018) developed nanofibers composed of Ir/IrO_2_ and RuO_2_ by using electrospinning and calcination. Optimizing the Ir-to-Ru ratio was helpful in controlling the morphology obtained. The catalyst had incredible HER activity in an alkaline environment, with an overpotential of 29.5 mV and a Tafel slope of 31.5 mV dec^−1^. Due to the direct generation of metallic Ir and Ru during cathodic polarization, which results in a mixed state between metallic and oxides states, the stability was also significantly enhanced. Hence, the dissolution problem arising from pristine oxides during the HER could be solved, indicating the importance of heterostructure MMO electrocatalysts.

## 4 Summary

We review the recent advances in heterostructured MMOs owing to their potential for use as HER electrocatalysts and identify the design strategies that hold promise for achieving high HER performance, which was evaluated based on the overpotential and Tafel slope values. The grain boundaries in heterostructured MMOs play an important role as a more exposed active site. The number of exposed active sites can easily be enhanced by increasing the number of heterojunctions. The heterojunction optimized the HER binding strength by combining two components from two different leg positions in the volcano plot. Heterostructured MMOs can also enhance the stability of metal oxides by altering the half-cell potential of the oxides. Therefore, MMOs are promising candidates for HER electrocatalysts because of their high HER activity, vast metal oxide choices, and various preparation strategies. Although many MMO catalysts have been reported with outstanding performances, challenges remain, such as the need for mechanistic understandings on active sites, due to the compositional diversity and dynamic change during the catalytic reaction. *In situ* analysis techniques, such as Raman spectroscopy and infrared spectroscopy, could reveal direct evidence of active sites of catalysts. In conjunction with spectroscopic approaches, computational science could also be applied to theoretically support the experimental results. This review provides a comprehensive understanding of the MMO design strategy for improving HER performance and provides new insights into the development of MMO interfaces.
